# AN EVALUATION OF REIMAGINE AGING: A NEW THEORY-BASED PROGRAM TO REDUCE INTERNALIZED AGEISM

**DOI:** 10.1093/geroni/igad104.2692

**Published:** 2023-12-21

**Authors:** Dallas Murphy, Michelle Porter, Corey Mackenzie

**Affiliations:** University of Manitoba, Winnipeg, Manitoba, Canada; University of Manitoba, Winnipeg, Manitoba, Canada; University of Manitoba, Winnipeg, Manitoba, Canada

## Abstract

Over the lifespan individuals may internalize ageist beliefs and as they enter older age, direct them towards themselves. This internalized ageism has many deleterious effects. Despite this, few theory-based interventions have attempted to decrease internalized ageism. As such, a six-week online program was developed including education, acceptance and commitment therapy, and attributional retraining to target mechanisms of change (psychological flexibility, mindfulness, perceived control, and empowerment). The six 90-minute sessions consisted of recorded videos, and discussion groups, with during and between-session activities also being part of the program. To evaluate the feasibility of this intervention, a sub-sample of 81 program participants (58 – 85 years old, 92% female) were sent an online questionnaire following each session. Each session received between 77 and 80 responses. Results were overwhelmingly positive. On a scale of 1 (not very useful) to 5 (very useful), roughly two-thirds (65%) rated the program as a whole very useful. Items participants felt the most important to learn included ageism information, acceptance and commitment therapy tools, and reimagining what it means to age well. Participant’s opinion on what they liked most about the program varied. Among others, common aspects identified were the informational videos, the activities, and the discussion groups. Roughly 81% of participants indicated that they completed the between-session activities, and 79% completed bonus activities. The vast majority indicated the program changed their views on ageism and/or internalized ageism. Going forward, we will evaluate this program’s ability to decrease internalized ageism, and the processes by which it achieves this.

